# Uterine rupture in patients with a history of hysteroscopy procedures: Case series and review of literature

**DOI:** 10.1097/MD.0000000000037428

**Published:** 2024-03-08

**Authors:** Liping Shao, Zhilong Yang, Huifang Yan, Rong Xu

**Affiliations:** a Department of Obstetrics and Gynecology, Changzhou Cancer Hospital Changzhou Fourth People’s Hospital, Changzhou, Jiangsu, China; b Department of General Surgery, Nanjing Lishui People’s Hospital, Nanjing, Jiangsu, China; c Department of Obstetrics and Gynecology, Nanjing Lishui People’s Hospital, Nanjing, Jiangsu, China.

**Keywords:** case reports, diagnosis, obstetric labor complications, review of literature, uterine rupture

## Abstract

**Rationale:**

Uterine rupture during pregnancy poses significant risks to both the fetus and the mother, resulting in high mortality and morbidity rates. While awareness of uterine rupture prevention after a cesarean section has increased, insufficient attention has been given to cases caused by pregnancy following hysteroscopy surgery.

**Patient concerns:**

We report 2 cases here, both of whom had a history of hysteroscopy surgery and presented with severe abdominal pain during pregnancy.

**Diagnoses:**

Both patients had small uterine ruptures, with no significant abnormalities detected on ultrasonography. The diagnosis was confirmed by a CT scan, which showed hemoperitoneum.

**Interventions:**

We performed emergency surgeries for the 2 cases.

**Outcomes:**

We repaired the uterus in 2 patients during the operation. Both patients recovered well. The children survived. No abnormalities were detected during their follow-up visits.

**Lessons:**

Attention should be paid to the cases of pregnancy after hysteroscopy.

## 1. Introduction

Uterine rupture is a rare but severe obstetric complication that significantly affects maternal and perinatal outcomes. It can occur due to various factors, with uterine scarring from cesarean sections and uterine-related surgeries being the most common causes. Ruptures during pregnancy are more critical than those during delivery, necessitating increased physician attention. Despite increased awareness of uterine rupture prevention after cesarean sections, prevention strategies for pregnancies following hysteroscopy remain insufficient. Therefore, this paper retrospectively examines 2 cases of spontaneous uterine rupture during pregnancy after hysteroscopy procedures at our hospital over the past 2 years.

## 2. Case report

### 2.1. Case 1

A 28-year-old woman (gravida 2, para 0), at 33 weeks and 2 days of gestation was brought to the hospital due to hypogastralgia for 13 hours. She underwent hysteroscopic separation of intrauterine adhesions at another hospital in 2017. Uterine perforation occurred during the operation and no repair was performed. At 33 weeks and 1 day of gestation, she experienced hypogastric pain on the right side, which gradually intensified and spread across her entire abdomen. A reexamination via B ultrasound revealed renal hydronephrosis with bilateral upper ureteral expansion, right kidney calculi, and encapsulated effusion in the low echo area in front of the urinary bladder. Obstetric ultrasound confirmed a singleton breech presentation with placenta previa. Upon physical examination, her temperature was 36.9°C, blood pressure was 137/90 mm Hg, and pulse rate was 99 bpm, but she exhibited abdominal distension, a tense abdominal wall, and significant abdominal pain, primarily on the right side. Blood tests showed a white blood cell of 11.25 10^9^/L, N of 84.4%, and hemoglobin of 86 g/L.

**Figure 1.**
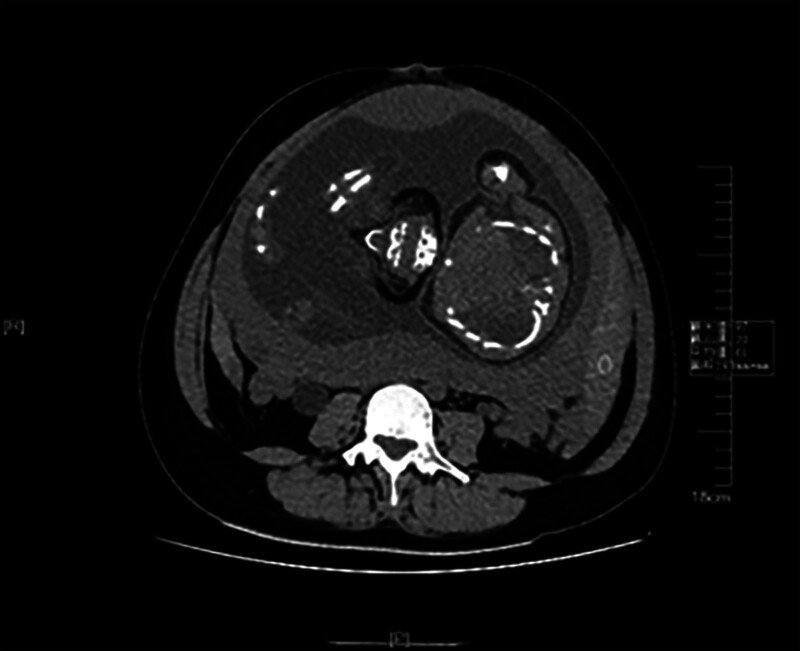
Computed tomography demonstrates pelvic and abdominal effusion.

Hypogastric pain was attributed to hydronephrosis, leading to the administration of magnesium sulfate for spasmolysis. However, her condition did not improve. Due to the long time waiting for a magnetic resonance imaging (MRI) appointment, after communication with patient and her family members for abdominal computed tomography (CT) examination was performed. A CT scan of the entire abdomen revealed abdominal and pelvic effusion and hematocele (Fig. 1).

**Figure 2.**
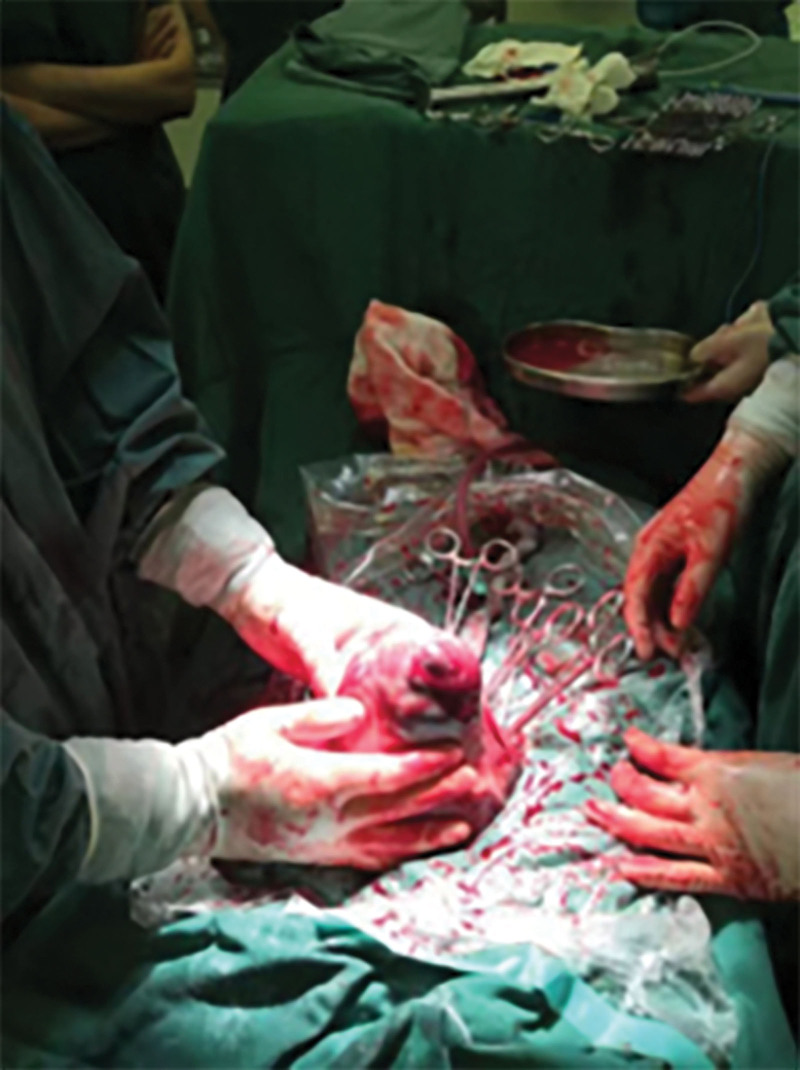
A bulge (1 × 1 cm) is observed in the wall of the right corner of the uterus.

Given her persistent hypogastric pain, evident peritoneal irritation signs, and unclotted blood extracted through hypogastric puncture under B-type ultrasound guidance, surgical intervention was performed with the patient’s and her family’s consent. During surgery, 800 mL of hematocele was found in her abdominal cavity, along with a 1 cm rupture at the right corner of her uterus (Fig. 2). Active hemorrhage was observed, and a live baby boy was delivered. Simultaneously, uterine repair was conducted during the surgery, which proved successful, ensuring the survival of both mother and baby. The newborn was transferred to the pediatric department. Six days post-surgery, the patient was discharged from the hospital, with no abnormalities noted during the 1-year follow-up visit.

### 2.2. Case 2

**Figure 3.**
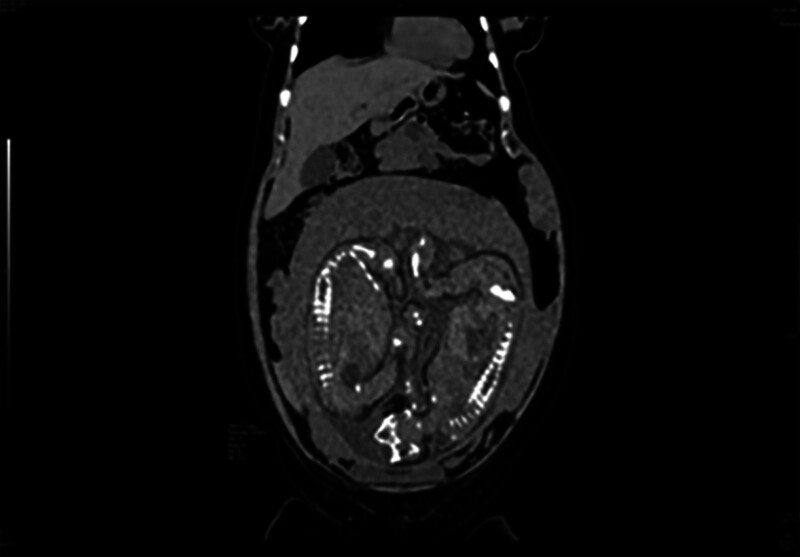
Computed tomography reveals a high probability of pelvic and abdominal hemoperitoneum.

**Figure 4.**
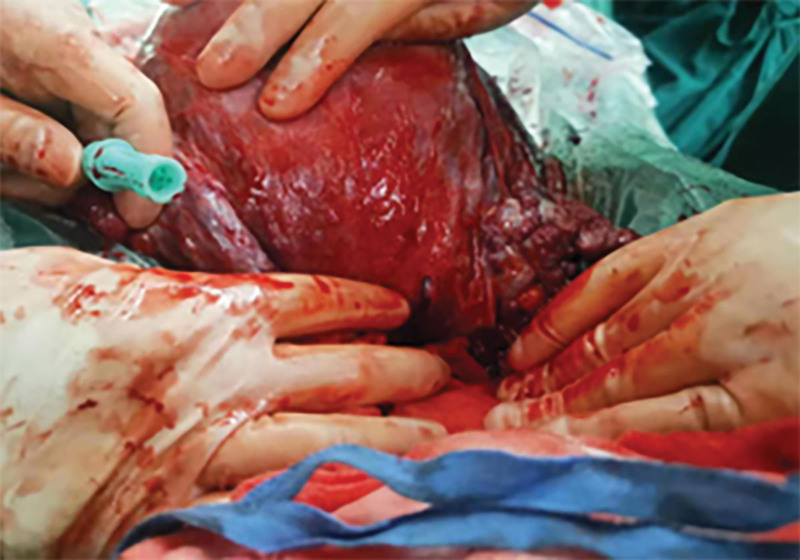
A rupture of 1 cm is observed at the lower of the posterior wall of the uterus.

On July 27, 2021, a 36-year-old woman (gravida 2, para 1, with a history of cesarean surgery) was admitted to the hospital due to “4-hour hypogastric pain at 32 weeks and 2 days of gestation.” Her surgical history included a cesarean section in 2006 and hysteroscopic separation of intrauterine adhesions in 2019. At 33 weeks and 2 days of gestation, she suddenly experienced persistent, stabbing hypogastric pain on the right side, without other discomfort. Her vital signs were stable, and she displayed mild pain, abdominal distension, and right lower abdominal tenderness. Obstetric ultrasound revealed a third-trimester twin pregnancy. Upon physical examination, her temperature was 36.9°C, blood pressure was 111/81 mm Hg, and pulse rate was 80 bpm, blood tests showed a white blood cell of 21.82 × 10^9^/L, neutrophil of 87.5%, and hemoglobin of 109 g/L. After hospitalization, dexamethasone was administered to accelerate fetal lung maturity, along with magnesium sulfate for spasmolysis therapy. Following consultation with the patient and her family, a CT scan of the entire abdomen was conducted, showing the possibility of right lower abdominal hemorrhage due to high-density images, multiple gallbladder stones, and a twin pregnancy (Fig. 3). Due to the presence of intraperitoneal hemorrhage, surgical intervention was performed with the patient’s and her family’s consent. During surgery, 300 mL of hematocele was discovered in her abdominal cavity, along with a cleft in the posterior wall of her uterus. Two live baby girls were delivered, and a 1 cm rupture was observed on the lower section of her uterine posterior wall, instead of the previous cesarean section scar location (Fig. 4). Uterine repair was carried out successfully during the surgery, ensuring the well-being of both the mother and the babies. Both infants were transferred to the pediatric department, and the patient was discharged from the hospital 6 days after the surgery. No abnormalities were detected during her follow-up visits.

## 3. Discussion

### 3.1. Clinical manifestation

A critical obstetric condition known as uterine rupture occurs when the body, bottom, or lower part of the uterus ruptures during delivery or late pregnancy, leading to severe hemorrhaging and posing a life-threatening risk to both the mother and the fetus.^[1]^

The typical clinical presentations of uterine rupture comprise abdominal pain, anomalous fetal heart rate monitoring, and vaginal bleeding.^[1]^ Abdominal pain stands as the most prevalent indication of uterine rupture and often functions as an early indicator, albeit lacking specificity. In clinical practice, differentiation from other conditions capable of eliciting abdominal pain, such as uterine contractions, gastrointestinal spasms, appendicitis, among others, becomes imperative.^[2]^ When positive tenderness is detected during abdominal examination, particularly uterine fundal tenderness, a strong suspicion of uterine rupture should arise. Some patients might undergo concealed rupture, where pain symptoms and signs do not immediately manifest. For instance, Nishikawa et al^[3]^ documented a case of uterine rupture transpiring during labor. Because the rupture was situated on the posterior wall and adhered to the ovaries and colon, hemorrhage was minimal. The patient experienced abdominal distension and discomfort nine days after delivery, and the rupture was only discerned through an MRI examination.

### 3.2. Etiology

The primary causes of uterine rupture encompass uterine scar rupture and obstructed labor. Uterine rupture at sites without prior cesarean section scars is relatively infrequent. The primary high-risk factors for uterine rupture at non-cesarean scar sites predominantly involve concealed scar uterus (involving a history of induced abortion and hysteroscopy surgery), fetal malposition, inadequate development of the uterine muscle layer, anomalous uterine development, external version procedures, multiparity, placental abnormalities, labor induction using drugs, fetal head disproportion, uterine adenomyosis, connective tissue disorders, a history of ectopic pregnancy, and laparoscopic cervical cerclage.^[1,4–6]^ Hysteroscopic surgery is increasingly common and serves an indispensable role in diagnosing and managing intrauterine diseases. Uterine rupture during pregnancy subsequent to hysteroscopic surgery constitutes a long-term complication of these procedures. Notably, hysteroscopic uterine septum resection surgery and uterine perforation during hysteroscopy stand as prominent risk factors for uterine rupture during pregnancy following hysteroscopic surgery.^[1]^

The use of hysteroscopy in gynecological surgery has become increasingly widespread, making hysteroscopic procedures more common.^[7]^ Aydeniz et al^[8]^ reported complication rates of 0.28% and 0.22%, respectively, in 13,600 hysteroscopic procedures performed in the Netherlands. Surgical hysteroscopy is associated with both perioperative and delayed complications.^[9]^ While many clinical studies have indicated that uterine rupture rarely occurs after laparoscopic myomectomy, there has been a growing number of reported cases of uterine rupture associated with this procedure in the past 2 decades.^[10–12]^ Factors such as incomplete uterine suturing techniques, inadequate hemostasis leading to hematoma formation, or excessive use of monopolar or bipolar electrocoagulation for hemostasis have all been linked to an increased risk of postoperative uterine rupture.^[13,14]^ Uterine healing and remodeling following childbirth involve unique processes. Pathological evidence of incomplete uterine rupture suggests an increase in collagen content within the lesion and a decrease in the muscle tissue component.^[15]^ In the first patient’s case, she had undergone one hysteroscopic adhesiolysis procedure. The second patient had undergone a cesarean section and hysteroscopic separation of uterine adhesions. Notably, the location of uterine rupture in the second patient differed from that of her previous cesarean delivery, occurring in the posterior wall of the uterus rather than the lower uterine segment’s anterior wall. This could be attributed to an increase in collagen content and a decrease in muscle tissue within the lesion, resulting in partial damage to the endometrium.

### 3.3. Auxiliary diagnostic method

Ultrasound examination is widely acknowledged as the preferred imaging method for assessing acute abdominal conditions in pregnant women, particularly in the context of identifying uterine rupture, particularly in patients with concealed or asymptomatic uterine rupture. Ultrasound facilitates continuous and dynamic monitoring and possesses a diagnostic discovery rate of 67% for uterine rupture.^[16]^ Nevertheless, its accuracy can be affected by various factors, such as the size of the uterine rupture site, gestational age, pelvic conditions, and the experience of the ultrasound operator. In one of our cases (case 1), obstetric ultrasound failed to reveal any significant abnormalities. In contrast, a urinary system ultrasound uncovered renal hydronephrosis, leading to a misdiagnosis of the patient’s abdominal pain as attributable to renal hydronephrosis. CT and MRI scans offer advantages due to their broader scanning range, allowing assessment of other abdominal and pelvic organs. This proves valuable in distinguishing between diverse emergency conditions like acute pancreatitis, gynecological emergencies such as ovarian torsion, and placental abruption. CT scans are faster than MRI, and contrast-enhanced scans aid in the easier identification of disrupted muscle layers. In urgent scenarios, CT scans offer a swifter evaluation of the abdominal condition in pregnant women, serving as a valuable clinical diagnostic tool or for the exclusion of certain diseases. The utilization of CT scans during pregnancy should not be entirely avoided when the benefits for the mother outweigh the theoretical radiation risks to the fetus.^[2]^ Recent American College of Obstetricians and Gynecologists guidelines also indicate that the use of CT during pregnancy should not be categorically discouraged. The potential benefits should be thoughtfully weighed against the risks when deemed clinically necessary. In acute illnesses, the theoretical advantage to the mother from an early and precise diagnosis may surpass the risks to the fetus.^[17]^ MRI provides high-resolution soft tissue imaging, is radiation-free, and can be employed in both prenatal and postnatal cases of uterine rupture. It enables multi-dimensional imaging of the uterine wall and surpasses ultrasound in the visualization of fetal organs and structures.^[18]^ Consequently, both CT and MRI scans can serve as vital supplementary tools for diagnosing and managing uterine rupture.

### 3.4. Prevention

Uterine rupture is a critical condition, and prompt surgical intervention is imperative to minimize adverse outcomes for both the mother and the baby. Consequently, early recognition holds paramount importance. Obstetricians should remain vigilant for classic symptoms and signs of rupture, including sudden and persistent abdominal pain, uterine tenderness, and unexplained fetal heart rate anomalies. In cases with atypical presentations, where patients might experience only abdominal discomfort or minor vaginal bleeding, a comprehensive evaluation, including a medical history review, physical examination, and auxiliary tests, becomes necessary. It is crucial to differentiate uterine rupture from other acute abdominal emergencies, such as appendicitis, pancreatitis, ovarian tumor torsion, and tumor rupture. Early diagnosis, even in cases with atypical symptoms or signs, and timely intervention can significantly reduce maternal complications and perinatal mortality rates, leading to improved pregnancy outcomes and prognosis. Delayed or misdiagnosis as other gynecological or non-gynecological conditions can result in fetal or neonatal mortality. The choice of surgical procedure hinges on the extent of uterine bleeding and the patient’s reproductive requirements. The primary objective is to expedite the fetus’s delivery during surgery. Surgical options may encompass uterine rupture repair, subtotal uterine resection, or total uterine resection. For patients in the early or mid-term of pregnancy, with minimal bleeding and a small rupture, and in the absence of severe infection, some researchers have recommended a combined approach of uterine rupture repair and elective cesarean section, thereby prolonging the pregnancy to a viable gestational age. These cases offer novel treatment strategies for clinical practice.^[19,20]^ There is no standardized surgical treatment for uterine rupture, and the choice of procedure may vary depending on the patient’s and fetal conditions.

### 3.5. Experiences and lessons

From the diagnosis and treatment of 2 atypical cases, we have summarized the following lessons: For pregnant women with concomitant surgical diseases, in addition to improving the diagnostic ability of ultrasound physicians, obstetricians should also inquire about medical history, carefully judge and differentiate. When considering the possibility of uterine rupture, CT and MRI can be used to clearly display the condition of the uterine wall, as well as the relationship between the uterus, fetus, and placenta. When there is intra-abdominal bleeding, abdominal puncture can be performed under ultrasound guidance to determine the nature of the fluid; atypical uterine rupture can easily lead to misdiagnosis and mistreatment by clinical doctors due to the atypical nature of its medical history, symptoms, signs, and examinations, resulting in serious consequences. Therefore, for pregnant women with a history of uterine cavity operations, including induced abortion, curettage, and salpingectomy, when the signs of uterine rupture are atypical, when sudden or progressive abnormal fetal heart rate monitoring occurs, comprehensive assessment of fetal heart rate, fetal movement, and maternal vital signs should be performed to avoid missed diagnosis and misdiagnosis, and active management should be carried out.

## 4. Conclusions

In conclusion, we report here 2 cases of uterine rupture during pregnancy after a history of hysteroscopic surgery, with uterine repair and successful delivery after emergency surgery. As the trend of delaying childbirth among women continues to grow, there is an increasing population of pregnant women with a history of gynecological surgery. Various surgical procedures result in diverse uterine scar locations, rendering the diagnosis of post-pregnancy secondary uterine rupture more intricate and demanding. This complexity is particularly evident in pregnant women lacking a history of uterine scarring. Hence, it is advisable to rigorously evaluate the criteria for gynecological surgeries and reduce unnecessary laparoscopic hysteroscopy-induced artificial abortions. Furthermore, it is recommended to initiate early prenatal assessments and consultations in obstetrics to exclude conditions like uterine anomalies, such as unicornuate uterus, bicornuate uterus, septate uterus, and poorly healed cesarean section scars. For pregnant women with a history of cesarean section or other high-risk factors for uterine rupture, obstetrics clinics should enhance educational efforts and management practices. This should involve delivering personalized care and formulating rational delivery plans. In instances where deemed necessary, the consideration of elective cesarean sections for pregnancy termination is prudent.

## Acknowledgments

We would like to express our gratitude to Mr. Zhufeng Xu from the Imaging Department for providing us with the visual images.

## Author contributions

**Formal analysis:** Liping Shao.

**Validation:** Liping Shao.

**Writing – review & editing:** Liping Shao, Rong Xu.

**Data curation:** Zhilong Yang, Huifang Yan.

**Investigation:** Zhilong Yang, Huifang Yan.

**Resources:** Zhilong Yang, Huifang Yan.

**Software:** Zhilong Yang, Huifang Yan.

**Visualization:** Zhilong Yang, Huifang Yan.

**Conceptualization:** Rong Xu.

**Funding acquisition:** Rong Xu.

**Methodology:** Rong Xu.

**Project administration:** Rong Xu.

**Supervision:** Rong Xu.

**Writing – original draft:** Rong Xu.
